# The Evaluation of Optic Nerves Using 7 Tesla “Silent” Zero Echo Time Imaging in Patients with Leber’s Hereditary Optic Neuropathy with or without Idebenone Treatment

**DOI:** 10.3390/jcm9041112

**Published:** 2020-04-13

**Authors:** Cezary Grochowski, Mark Symms, Kamil Jonak, Paweł Krukow, Tobias C Wood, Emil Ljungberg, Joaquim Enseñat, Katarzyna Nowomiejska, Robert Rejdak, Ryszard Maciejewski, Gareth J. Barker

**Affiliations:** 1 Department of Anatomy, Medical University of Lublin, Lublin, 20-400 Lublin, Poland; maciejewski.r@gmail.com; 2 Department of Neurosurgery, Hospital Clínic de Barcelona, University of Barcelona, 08036 Barcelona, Spain; JENSENAT@clinic.cat; 3 Department of Neurosurgery, Medical University of Lublin, 20-954 Lublin, Poland; 4 General Electric (GE) Healthcare, Amersham Place, Amersham HP7 9NA, UK; MarkRoger.Symms@ge.com; 5 Department of Psychiatry, Psychotherapy and Early Intervention, Medical University of Lublin, 20-439 Lublin, Poland; jonak.kamil@gmail.com; 6 Department of Biomedical Engineering, Lublin University of Technology, 20-618 Lublin, Poland; 7 Department of Clinical Neuropsychiatry, Medical University of Lublin, 20-439 Lublin, Poland; pawelkrukow@umlub.pl; 8 Department of Neuroimaging, King’s College London, London WC2R 2LS, UK; tobias.wood@kcl.ac.uk (T.C.W.); emil.ljungberg@kcl.ac.uk (E.L.); gareth.barker@kcl.ac.uk (G.J.B.); 9 Department of General Ophthalmology, Medical University of Lublin, 20-400 Lublin, Poland; katarzynanowomiejska@lycos.com (K.N.); robertrejdak@yahoo.com (R.R.)

**Keywords:** 7T MRI, 7 tesla, ultra high field MRI, silent, Zero Echo Time, Leber, neuropathy, optic nerve, magnetic resonance

## Abstract

Magnetic Resonance Imaging (MRI) of the Optic Nerve is difficult due to the fine extended nature of the structure, strong local magnetic field distortions induced by anatomy, and large motion artefacts associated with eye movement. To address these problems we used a Zero Echo Time (ZTE) MRI sequence with an Adiabatic SPectral Inversion Recovery (ASPIR) fat suppression pulse which also imbues the images with Magnetisation Transfer contrast. We investigated an application of the sequence for imaging the optic nerve in subjects with Leber’s hereditary optic neuropathy (LHON). Of particular note is the sequence’s near-silent operation, which can enhance image quality of the optic nerve by reducing the occurrence of involuntary saccades induced during Magnetic Resonance (MR) scanning.

## 1. Introduction

The Optic Nerve Sheath Complex (ONSC) is a structure of the central nervous system, considered radiologically to consist of the optic nerve, the surrounding three meningeal layers, and cerebrospinal fluid (CSF). Its axons are myelinated by oligodendrocytes.

The ONSC is of wide-ranging clinical interest because many pathologies are located in it or are associated with it. The ONSC is small, its cross-section occupying very few pixels of a typical magnetic resonance (MR) image, but it is also extended, curving in three-dimensions. It is difficult to obtain high quality MR Images of both this structure and the brain in a clinically acceptable time [1]. The optic nerve and the eyeball have been shown to frequently move considerable distances (of the order of a cm) during a relatively short (three-minute) scan, even in healthy volunteers who are fixating on an external landmark. Motion and pulsatile effects cause ghosting and other artefacts in the phase-encoding direction of conventional MR images.

The optic nerve is situated near head structures—the skull base and the air-filled sinuses—which cause large B1+ and B0 inhomogeneities. “Snapshot” techniques such as echo planar imaging (EPI), often used to freeze motion, are susceptible to geometric distortions in these regions. Many subjects also have metallic implants (e.g., dental) in this region.

Optic nerve imaging protocols usually include multi-slice T1 and T2-weighted scans in both axial and coronal orientations, as well as post-contrast scans [2,3]. Partial volume effects in two-dimensional sequences, however, often compromise accurate depiction of the optic nerves along their full extent.

The scan time of each sequence is customarily kept short to reduce motion artifact. Yiannakis et al. took further steps to address optic nerve motion by acquiring a series of quick two-dimensional images, allowing the subject to relax between periods of fixation, and then applying in-plane registration as a post-processing step [4].

Three-dimensional volumetric sequences are longer in duration, and should consequently suffer higher motion degradation, but some groups have obtained reliable results using T1-weighted spoiled gradient-recalled sequence (SPGR) [5] or a heavily T2-weighted 3D slab [6].

In the quest for more specific pathologic information, other image contrasts of the optic nerve have also been acquired. Diffusion has been used as a measure of axon integrity, though the acquisition is usually done with an EPI-based sequence, so geometric distortion and signal-to-noise ratio (SNR) both limit the achievable image resolution [7]. Wheeler-Kingshott et al. acquired multiple averages of each diffusion encoding direction (>40) rather than the High Angular Resolution Diffusion Imaging (HARDI)many-direction diffusion schemes preferred for brain diffusion tensor imaging (DTI) [8]. Using such techniques, they have observed that the optic nerve moves significantly during image acquisition [9].

A Magnetization Transfer Ratio (MTR) has also been used, especially in the study of optic neuritis [10]. The literature currently has differing conclusions about whether MTR is sensitive to demyelination (compare Kolappan [11] and Klistorner [12]). It seems more certain that MTR changes correlate with retinal nerve fiber layer (RNFL) thickness [13] and a loss of visual acuity.

Leber’s hereditary optic neuropathy is a genetically inherited disease of the optic nerve. Theodor Leber described the disease for the first time in 1871 [14]. In his study Leber analyzed symptoms in four families and discovered that the pathology occurs almost exclusively within the optic nerve. Moreover, he described centrally-occurring scotoma, rapid disease progression within 2–4 weeks (after this time no disease progression is observed or it is very slow) and accompanying symptoms in some patients such as arrhythmias as well as consecutive optic nerve involvement, which was later confirmed in several different studies [15,16,17,18]. In addition, Leber noted the co-occurrence of color blindness, which was also retreating in patients whose vision had improved over time. From a genetic standpoint, LHON is the first disease associated with maternal inheritance and associated with a point mutation in mitochondrial DNA [19,20]. The disease usually affects men, and is associated with degeneration within the retinal ganglion cells and loss of axons within the optic nerve. More than 95% of Leber’s hereditary optic neuropathy cases are caused by point mutations within three mitochondrial DNA: T14484C, G3460A, and G11778A, which encode complex I of the respiratory chain subunit.

Current literature does not provide a complete picture about the abnormalities associated with LHON that can be visualized using magnetic resonance imaging. The short tau inversion recovery (STIR) protocol is commonly used for imaging optic nerve disease such as optic neuritis, allowing good depiction of the inflammation within the optic nerve as well as due to its short acquisition time [21]. There are several case reports describing abnormalities in MRI in the anterior optic pathway region. Kermode et al. reported the occurrence of hyperintensities within the posterior part of the optic nerve in 13 subjects [22] and several studies have reported hyperintensities within the anterior part of the optic nerve [23,24,25,26,27,28]. Recent papers, in which high-field magnetic resonance was used, have reported hyperintense areas within the distal parts of the optic nerve, optic chiasm, optic tract, and enlargement of the optic chiasm.

In two cases of LHON, increased signal on T2-weighted scans was seen not only in the optic nerve but also in the optic tracts, extending as far as the lateral geniculate bodies [29,30]. Chiasmal enlargement and optic nerve enhancement was observed in Gadolinium-enhanced T1-weighted imaging in two cases of Leber’s hereditary optic neuropathy [31]. T2-weighted MRI showed hyperintensity in the posterior portion of both optic nerves and in the optic chiasm in 19 of 28 LHON patients, and enlargement of the chiasm was found in 16 patients [32]. In a case of LHON, T2-weighted fluid-attenuated inversion recovery (FLAIR) imaging revealed asymmetrical chiasmal hyperintensities and enlargement and chiasmal enhancement on Gadolinium-enhanced T1-weighted imaging [33].

Notably, two groups have performed quantitative MRI on the optic nerve in LHON. Inglese et al. showed that optic nerve volume, mean diffusion and Magnetization Transfer Ratio measures were changed in LHON [34]. Iorga et al. showed that optic nerve volumes measured on 3D T1-weighted images were lower in two familial cases of LHON [35].

It is possible that some of the variability of findings in these case reports could be explained by the challenges of imaging the optic nerve at 1.5T in the clinic. Many workers have reported the superiority of 3T MRI over 1.5T MR due to better spatial resolution and higher SNR. Mafee et al. used thin-section high-resolution spin-echo T2-weighted imaging to evaluate orbital structures as well as intracranial pathologies [36]. There are 7-T MR systems are now installed at many sites and they offer the promise of higher sensitivity and image resolution for MRI of the brain and related structures.

One recent work has demonstrated the use of Silent imaging (their term) at 7 tesla (7T) for the eye [37]. They used a topical coil that only gathered a strong signal from the eye, but the images show promising details in the optic nerve. The authors also asserted that the Spectral Presaturation with Inversion Recovery (SPIR) fat saturation pulse used in their sequence gives Magnetization Transfer contrast.

Symms et al. reported Silent 7T images [38] in which the Adiabatic Spectral Inversion Recovery (ASPIR) fat suppression pulse also gave Magnetisation Transfer contrast. The images had high radiofrequency (RF) power deposition, were necessarily of short scan duration (<1 min) and were of moderate nominal voxel size (1.4 mm). Grey/white contrast and fat suppression, particularly in the orbits, were considered excellent.

We have developed a high-resolution version of this sequence (dubbed “Silent- Magnetization Transfer (MT)”) which has clinically acceptable levels of RF power deposition, but otherwise retains good G/WM contrast and excellent fat suppression, including in the region containing the orbits. Scan time is short (2 min). During operation, the sequence is almost completely free of acoustic noise, which explains the sequence name of “Silent-MT”.

Overall, the presented data suggest that is there is still a need to develop and verify new methods enabling precise and clinically effective visualization of the Optic Nerve Sheath Complex structures. On the other hand, imaging literature regarding the state of the visual system in LHON might be considered mixed and incomplete. Having this in mind, the major goal of this study was to obtain ONSC 7T images based on the Silent-MT protocol of the new sample of Polish LHON patients, who had never previously participated in a neuroimaging study. Additionally, referring to the LHON pathophysiology [33,34] for which the asymmetry of optic nerve atrophy is typical, we conducted a quantitative analysis of the right and left optic nerve formations in a patient group to verify whether applied Silent-MT protocol enables us to confirm such a potential asymmetry. There are some studies [39,40], including completed clinical trials [41], suggesting that several symptoms of LHON might be improved with Idebenone therapy. Because some of our patients were also treated with this preparation, an additional goal was to compare selected features of the ONSC between patients treated and untreated with Idebenone. Although subgroups created on the basis of the treatment criterion contained a relatively small number of cases, possible significant differences between them might be considered as an additional rationale for using Idebenone in the therapy of Leber’s disease.

## 2. Materials and Methods

### 2.1. Silent-MT Sequence

The sequence is a 3-dimensional version of the sequence RUFIS, first introduced by Madio and Lowe [42]. The signal is sampled after a hard RF pulse, which is applied in the presence of an imaging gradient and this unit (called a “spoke”) is repeated while the spatial orientation of the imaging gradient is slowly varied. K-space is sampled in a radial manner, with each spoke oriented in a slightly different direction. The small gradient changes between spokes make very little acoustic noise, even with the higher motor forces associated with the 7-Tesla field. A train of spokes is commonly referred to as a segment. Image reconstruction can be performed using algorithms similar to those used for Projection Reconstruction imaging techniques.

The delay between the RF pulse and the signal sampling period can be made very short—of the order of 20 ms. This sequence is typically, though perhaps not accurately, classified as a ZTE (Zero Echo Time) sequence.

This low acoustic noise imaging sequence is called “Silent” and, to our knowledge, is available on two of the three major MR scanner platforms [41,43].

In this study, 192 spokes were acquired; at the start of each group of spokes, an ASPIR pulse was applied. The ASPIR pulse is part of a family of pulses that suppress fat by selectively inverting the fat signal [44] while leaving the water signal undisturbed. A delay (of about 100 ms) allows fat magnetization time to cross zero as it recovers from the inversion; if the imaging sequence is applied at approximately this point in time, the resulting images are observed to be almost completely free of contribution from fat.

Selective Spectral Inversion pulses are necessarily applied near the fat resonance frequency instead of the water resonance. At 7T the fat-water shift is approximately 1026 Hz. The ASPIR pulse in this study was applied at an offset of 1250 Hz from the water resonance. This offset is in the frequency range that is typically used to generate significant Magnetization Transfer (MT) effect; for instance Helms et al. used an offset of 2.2 kHz [45].

Previously, we demonstrated Magnetisation Transfer contrast in Silent with an ASPIR pulse applied once every 32 spokes [41]. However, the frequent application of the ASPIR pulse made the sequence time-inefficient and resulted in high RF power deposition, which limited scan time and the resolution which could be achieved.

### 2.2. Participant Population

Fifteen participants (male: female = 13:2) who have been diagnosed with Leber’s hereditary optic neuropathy and presented confirmed 11778G>A mitochondrial DNA mutation were included. The mean age of the participants was 36.2 years (range: 18–67 years). Five of the participants were over 40 years old. Six patients received the Idebenone therapy (the recommended dose was 900 mg idebenone per day, with a dose of two 150-mg tablets three times a day). The mean age of patients with Idebenone therapy was 32, the two females were receiving the therapy. Beside the LHON sample, one healthy volunteer (male, age 39) was also included to obtain images enabling qualitative comparison of 7-T Silent-MT results gained from the patients and the unaffected individual. Blood pressure was measured in all participants, and no abnormalities were found. All participants signed informed consent. The study included participants who had no known pathology within the cerebrovascular system, were capable of signing informed consent, were over 18 years old, and had a diagnosis of LHON confirmed by genetic tests. Patients with any metallic implant, who were pregnant or breastfeeding, as well as those suffering from claustrophobia, were excluded from this study. This research was approved by the local medical ethics committee of the Medical University of Lublin (KE-0254/23/2017) and was carried out in compliance with national legislation and the Declaration of Helsinki. The scans were obtained in 2019 at the Ecotech Complex, Lublin, Poland.

### 2.3. Radiological Assessment

The images were evaluated by an experienced neuroradiologist (25 years of experience), a neuroanatomy specialist (40 years of experience) and a neurosurgeon (5 years of experience) for appearance of the optic nerve (and surrounding structures) interpreted in light of the subjects’ conditions (healthy volunteer and LHON). There was 93% similarity in evaluation between the mentioned researchers.

### 2.4. Image Analysis

**Figure 1:**
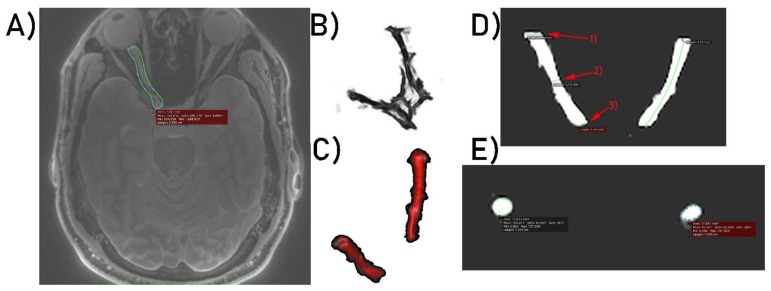
(**A**) Optic nerve segmentation area. (**B**) 3D model of both optic nerves. (**C**) 3D optic nerves’ reconstruction after unwanted brain tissue removal. (**D**) Optic nerve dimensioning process at three selected points, respectively: (1) 1st point of interest, (2) 2nd point of interest, (3) 3rd point of interest. (**E**) Calculation of optic nerves’ surface areas.

Acquired images in Digital Imaging and Communications in Medicine (DICOM) format were imported into OsiriX Lite software (OsiriX). The first step of post-processing was to perform manual optic nerve segmentation. This was achieved by manually segmenting the nerves slice-by-slice using the region of interest (ROI) and Repulsor tools (see Figure 1A). After the segmentation process was completed, 3D models of both optic nerves were created (Figure 1B). Using the “scissors” option, in 3D reconstruction mode in OsiriX, the remaining unwanted brain tissue was removed (Figure 1C). The last stage of the post-processing analysis was to measure the nerve length and its dimensions with the nerve cross-sectional surface areas at three different points localized on the optic nerve. Measurement points were placed as follows: the first point of measurement was placed on the optic nerve proximally to the eyeball, the second point was placed in the middle of the optic nerve and the final, third point was placed proximally to the optic chiasm (Figure 1D). Nerve length and dimensions as well as the nerve cross-sectional surface areas (Figure 1E) were calculated by two independent observers (neuroradiologist and neuroanatomy specialist) using the OsiriX built-in standardized functions.

## 3. Results

### 3.1. Radiological Inspection

**Figure 2:**
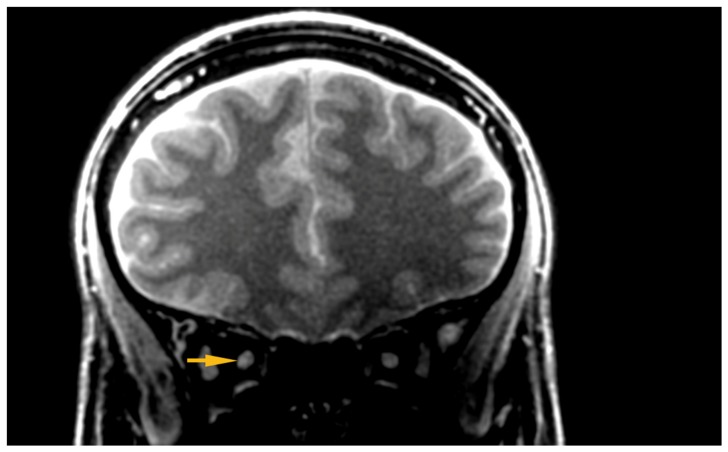
Hyper intensive area visible within the ONSC. ONSC, Optic Nerve Sheath Complex.

**Figure 3:**
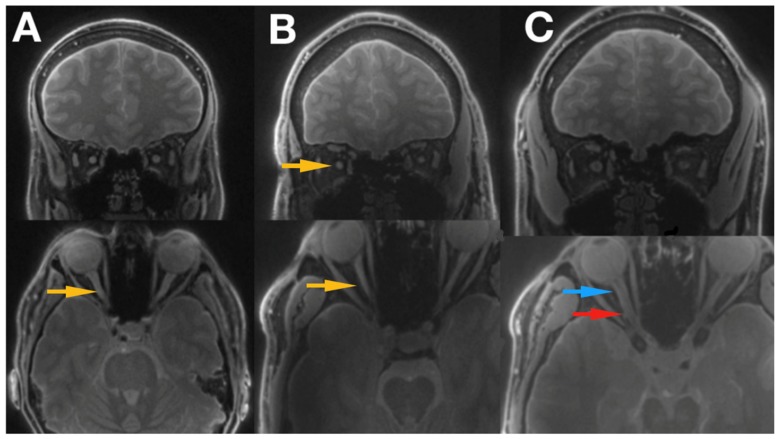
Shows the images of 2 patients with LHON disease ((**A**) Idebenone treatment, (**B**) no treatment received) and a healthy control subject (**C**). The optic nerve can be easily traced all the way to the optic chiasm, and optic tract is also clearly visible. Blue arrow indicates proximal part of the optic nerve, red arrow the distal part of the optic nerve, and yellow arrows the hyper intensive areas within the optic nerve. LHON, Leber’s hereditary optic neuropathy.

Silent-MT images obtained in a healthy control clearly distinguish the axonal part of the optic nerve and surrounding structures. The optic nerve is clearly visible as well as the surrounding CSF and the optic nerve sheath in the healthy individual. Silent-MT images showed good signal and strong contrast in the optic nerve, had excellent suppression of the fat surrounding the ONSC, and showed no motion artifact. In LHON patients hyperintense areas are visible along the whole optic nerve (Figure 2 and Figure 3). Moreover, the nerve itself looks thinner, with an irregular shape and structure. Abnormalities are mostly visible within the proximal and distal part of the optic nerve as well as within the optic disc area. Moreover, in this work we demonstrate that increasing the number of spokes per group to 192 increases the sequence time efficiency and reduces RF power deposition while maintaining significant MT contrast. There were no adverse events reported in any participant after the scanning.

### 3.2. Quantitative Analysis 

**Figure 4:**
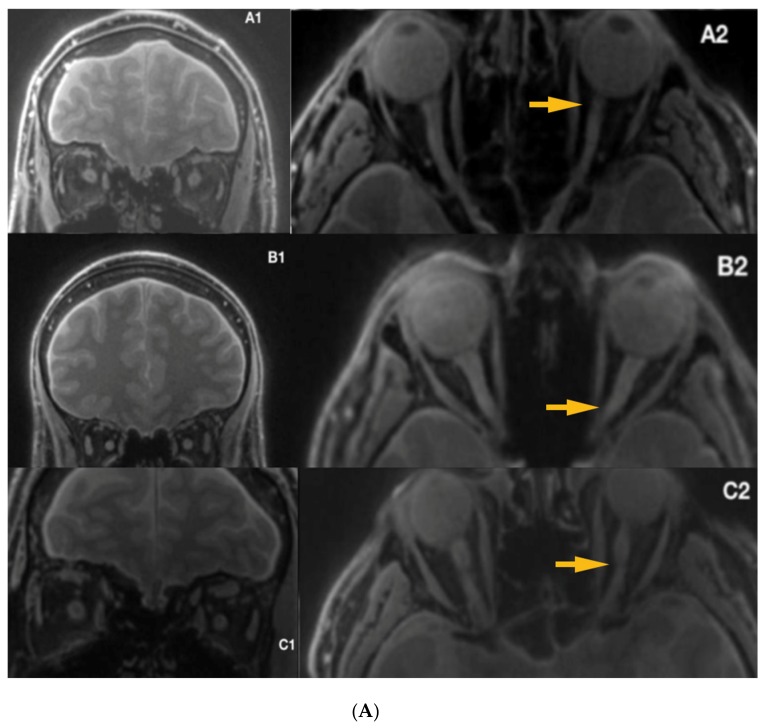

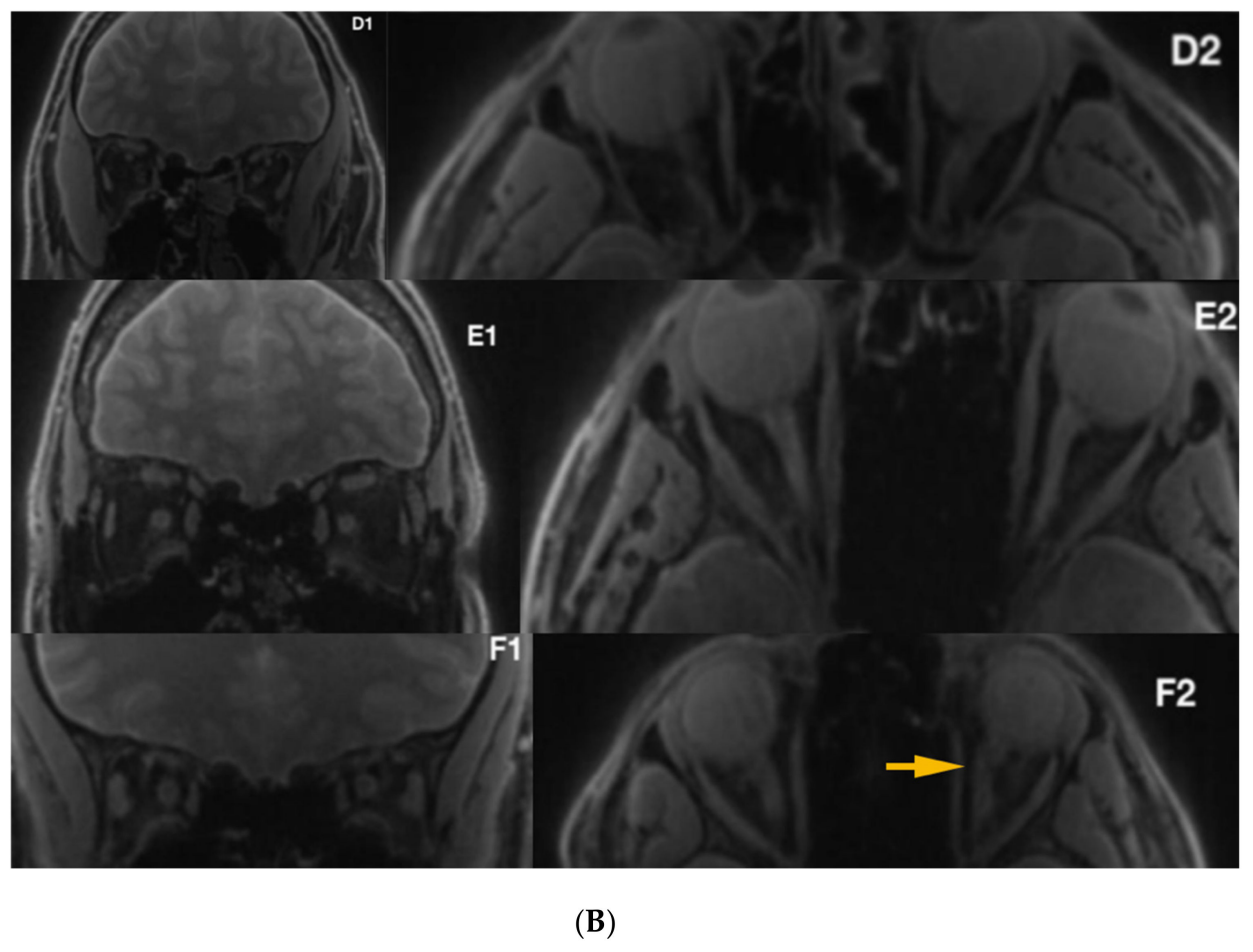
(**A**) Silent-MT images in 3 LHON subjects who received Idebenone therapy. The yellow arrows show irregularity within the optic nerve. (**B**) Silent-MT images in three LHON subjects who received Idebenone therapy. Optic nerve atrophy is not visible in Patient F, (disease duration more than one year), however the yellow arrow shows more tortuous anatomy of the optic nerve, which is consistent with the side of the disease. MT, Magnetization Transfer.

Mean duration of illness in LHON was 12.66 years (1-year minimum, 41 years maximum) and six of the patients (40%) (Figure 4) were treated with Idebenone (the duration of the treatment ranged from 8 months to 15 months). Due to the relatively small sample and quantitative characteristics incompatible with normal distribution, to compare subgroups, a non-parametric Mann–Whitney test (Z) was applied with *p* < 0.05 set as a statistical significance threshold. Each variable was individually compared between subgroups. Analysis of quantitative dimensions (length, diameters, and surface areas) for left and right optic nerves revealed statistically significant differences for nerve diameter and the surface area measured at the second point (*p* < 0.05); both parameters were larger for the right nerve (Table 1).

There were no statistically significant correlations between age, duration of illness, and any of the analyzed quantitative measures of nerve anatomy (all *p* > 0.05). A subgroup of patients receiving Idebenone had significantly larger diameter and surface area of the left nerve at the third measuring point compared with the untreated subgroup (Table 2). These subgroups were similar with respect to age, sex, and duration of illness (*p* > 0.05).

## 4. Discussion

In this paper, Magnetisation Transfer-weighted Silent (“Silent-MT”) images were evaluated for assessment of the optic nerve and surrounding structures in a cohort of subjects with Leber’s hereditary optic neuropathy. Silent-MT images showed good signal and strong contrast in the optic nerve, had excellent suppression of the fat surrounding the ONSC, and showed no motion artifact. Radiological inspection of Silent-MT images revealed hyper-intense areas along the optic nerve. 

Quantitative measures of segmented ONSC structures revealed significant changes in nerve diameter and cross-sectional area between left and right optic nerves in the Leber’s hereditary optic neuropathy cohort. The distal part of the left optic nerve was found to be significantly larger in Idebenone-treated patients; this finding in the distal area of the optic nerve was in agreement with several previously published reports [30,31,32,33,34].

Idebenone, a short-chain analogue of ubiquinone, has an ability to shuttle electrons from complexes I and II to complex III. Clinical trials proved the visual benefit after Idebenone treatment and suggest that when used within the time window, it may prevent the retinal ganglion cell death [29,30,31,32,46,47,48,49,50,51,52]. Disease onset among LHON carriers is characterized by acute or subacute painless loss of central vision, which is unilateral at the beginning; the other eye is usually affected within six to eight weeks, which may suggest more advanced abnormalities within the single starting nerve [53]. Our results, obtained with Silent-MT imaging, show significantly larger diameter and surface area of the left nerve at the third measuring point (distal part of the optic nerve), compared to the group not receiving the treatment. Previous research papers reported the presence of hyperintense areas within the distal part of optic nerves [29,50,51,52]. Although the duration of illness among analyzed patients is one year at minimum, (post-acute phase of the disease) there were hyperintense lesions visible within the optic nerves in acquired images, which could suggest the degeneration of the optic nerve as a late complication of the disease.

Silent is a 3-dimensional sequence radial sequence which has relatively low susceptibility to geometric distortions induced by static field inhomogeneities, even at 7T, so it is well-suited to imaging demanding areas of the head like the ONSC. We used a version of Silent which had previously been optimized for good image quality at 7T [38]. The radial acquisition of Silent does not encode spatial information in the signal phase, so ghosting artifacts related to phase-encoded aliasing of motion and cardiac pulsation do not occur. 

The Silent scan produces very little acoustic noise, which can improve subject comfort and compliance [47,48]. This reduces the probability of artifacts caused by subject motion.

Most imaging strategies to image the ONSC have used fast sequences (EPI, Fast/Turbo Spin Echo) which make loud noises. For the volunteer lying in the scanner, the subjective experience is that these loud noises have a “stereo headphones” effect: the scan sounds seem to arise from different locations within the scanner bore, and the locations of apparent noise source varies as the scan proceeds. It therefore seems reasonable to conclude that some of the eye motion observed during optic nerve imaging is caused by the loud noises generated by the fast MR sequences, and that the use of loud, fast MR sequences to “freeze” eye motion may actually be counter-productive. Silent sequences, with their very quiet operation, offer a unique advantage for this application. We note that Beenakker et al. report that they have observed more eye blinks during acoustically loud sequences [37].

Although our study has unequivocal advantages associated with a successful application of Silent-MT protocol to assess the state of optic nerves in LHON subjects demonstrating asymmetry of nerves atrophy and probable effective impact of Idebenone treatment on the progression of anatomical changes resulting from Leber’s neuropathy, it has some limitations which should be addressed in needed replication studies. It would be beneficial to compare images and quantitative metrics based on Silent-MT imaging with other imaging strategies applied to visualize the structures of the optic nerve, and, in the case of the clinical sample, to analyze possible correlations between anatomical results with the functional impairments of patients. Verification regarding such relationships would additionally help in assessing the neuroimaging method used in our research in terms of its clinical usefulness.

## 5. Conclusions

We used Silent-MT to image the Optic Nerve of a cohort of patients with Leber’s hereditary optic neuropathy at 7T. High quality, artifact-free images were obtained. The images showed changes in the optic nerve consistent with previous findings of this pathology, and quantitation of the optic nerve also yielded significant findings. It is possible that Silent-MT generates superior images because its silent operation does not induce saccades in the subject.

Silent-MT at 7T provides high-resolution, high contrast artifact-free Magnetisation Transfer-weighted images of the Optic Nerve in a short scan time, facilitating radiological inspection and quantitation.

## Figures and Tables

**Table 1:** Quantitative comparisons of the left and right optic nerves at three measurement points. This analysis concerns the entire group of patients with Leber’s disease (*n* = 15).

	Left		Right			
	M (SD)	Min–Max	M (SD)	Min–Max	Z	*p*
Length (cm)	4.31 (0.49)	3.50–5.50	4.28 (0.39)	3.70–5.20	0.59	0.550
Diameter (mm) 1	3.89 (0.63)	2.30–4.90	4.06 (0.81)	2.70–5.10	0.62	0.532
Diameter 2	1.95 (0.55)	0.70–3.00	2.32 (0.55)	0.90–3.10	2.03	**0.043**
Diameter 3	3.19 (0.39)	2.50–3.80	3.24 (0.31)	2.80–3.80	0.85	0.394
Surface area (mm^2^) 1	12.23 (3.84)	3.80–17.66	13.50 (5.23)	5.55–21.58	0.91	0.364
Surface area 2	3.27 (1.74)	0.39–7.48	4.56 (1.91)	0.64–7.95	2.04	**0.040**
Surface area 3	8.05 (1.92)	4.44–11.79	8.35 (1.64)	6.41–11.52	0.62	0.532

M, median; SD, standard deviation; Z, z-score.

**Table 2:** Comparison of quantitative nerves parameters in two LHON subgroups created on the basis of Idebenone treatment criterion.

	Idebenone Treatment (*n* = 6)		Without Treatment (*n* = 9)			
	M (SD)	Min–Max	M (SD)	Min–Max	Z	*p*
L. Length (cm)	4.28 (0.43)	3.50–4.70	4.33 (0.55)	3.60–5.50	0.02	0.968
L. Diameter (mm) 1	3.75 (0.82)	2.30–4.40	3.98 (0.51)	3.20–4.90	0.05	0.952
L. Diameter 2	2.06 (0.77)	0.70–3.00	1.87 (0.39)	1.30–2.50	−1.00	0.315
L. Diameter 3	3.47 (0.38)	2.90–3.80	3.01 (0.28)	2.50–3.40	−2.06	**0.038**
L. Surface area (mm^2^) 1	11.66 (4.79)	3.80–16.59	12.61 (3.33)	7.52–17.66	0.05	0.953
L. Surface area 2	3.96 (2.36)	0.39–7.48	2.81 (1.11)	1.33–4.75	−1.12	0.262
L. Surface area 3	9.47 (1.28)	7.18–11.79	7.11 (1.38)	4.44–8.66	−2.06	**0.038**
R. Length (cm)	4.16 (0.30)	3.70–4.60	4.36 (0.44)	3.80–5.20	0.94	0.345
R. Diameter (mm) 1	3.96 (0.75)	2.70–4.80	4.12 (0.88)	2.80–5.10	0.53	0.595
R. Diameter 2	2.38 (0.54)	1.50–3.10	2.27 (0.58)	0.90–3.00	−0.17	0.859
R. Diameter 3	3.35 (0.32)	2.80–3.80	3.17 (0.31)	2.90–3.80	−1.00	0.316
R. Surface area (mm^2^) 1	12.54 (4.35)	5.55–17.16	14.14 (5.90)	6.75–20.58	0.76	0.443
R. Surface area 2	4.76 (2.07)	1.73–7.95	4.42 (1.90)	0.64–7.75	−0.41	0.679
R. Surface area 3	8.85 (1.65)	6.63–11.35	8.02 (1.64)	6.41–11.52	−1.00	0.316

LHON, Leber’s hereditary optic neuropathy; L., left optic nerve; R., right optic nerve.
